# Improving 3D-printing of megavoltage X-rays radiotherapy bolus with surface-scanner

**DOI:** 10.1186/s13014-018-1148-1

**Published:** 2018-10-19

**Authors:** Giovanna Dipasquale, Alexis Poirier, Yannick Sprunger, Johannes Wilhelmus Edmond Uiterwijk, Raymond Miralbell

**Affiliations:** 1 Department of Radiation Oncology, Geneva University Hospital, CH-1211, 14 Geneva, Switzerland; 2 Medical School, Geneva University, Geneva, Switzerland; 3 IT consultant, Geneva, Switzerland; 4 Faculty of Medicine, Geneva University, Geneva, Switzerland

**Keywords:** Surface-scanner, 3D printing, Bolus, Radiotherapy, Additive materials, DIBH

## Abstract

**Background:**

Computed tomography (CT) data used for patient radiotherapy planning can nowadays be used to create 3D-printed boluses. Nevertheless, this methodology requires a second CT scan and planning process when immobilization masks are used in order to fit the bolus under it for treatment.

This study investigates the use of a high-grade surface-scanner to produce, prior to the planning CT scan, a 3D-printed bolus in order to increase the workflow efficiency, improve treatment quality and avoid extra radiation dose to the patient.

**Methods:**

The scanner capabilities were tested on a phantom and on volunteers. A phantom was used to produce boluses in the orbital region either from CT data (resolution ≈1 mm), or from surface-scanner images (resolution 0.05 mm). Several 3D-printing techniques and materials were tested. To quantify which boluses fit best, they were placed on the phantom and scanned by CT. Hounsfield Unit (HU) profiles were traced perpendicular to the phantom’s surface. The minimum HU in the profiles was compared to the HU values for calibrated air-gaps. Boluses were then created from surface images of volunteers to verify the feasibility of surface-scanner use in-vivo.

**Results:**

Phantom based tests showed a better fit of boluses modeled from surface-scanner than from CT data. Maximum bolus-to-skin air gaps were 1-2 mm using CT models and always < 0.6 mm using surface-scanner models. Tests on volunteers showed good and comfortable fit of boluses produced from surface-scanner images acquired in 0.6 to 7 min. Even in complex surface regions of the body such as ears and fingers, the high-resolution surface-scanner was able to acquire good models. A breast bolus model generated from images acquired in deep inspiration breath hold was also successful. None of the 3D-printed bolus using surface-scanner models required enlarging or shrinking of the initial model acquired in-vivo*.*

**Conclusions:**

Regardless of the material or printing technique, 3D-printed boluses created from high-resolution surface-scanner images proved to be superior in fitting compared to boluses created from CT data. Tests on volunteers were promising, indicating the possibility to improve overall radiotherapy treatments, primarily for megavoltage X-rays, using bolus modeled from a high-resolution surface-scanner even in regions of complex surface anatomy.

## Background

Superficial cancer lesions are difficult to irradiate because of megavoltage X-ray radiation physics (build-up effect). Therefore, extra material is added on top of superficial targets to achieve better tumor irradiation. This added material, called bolus, is used in radiotherapy to increase the dose and coverage of superficial tumors [1]. Often bolus design is a digital process that is entirely done using computers within a treatment planning system. However, the creation of a personalized bolus is still done by hand usually with thermoplastic materials like wax, which requires time and effort. Also, the result is not always satisfying in terms of fitting and reproducibility of daily positioning on the patient and radiation coverage of the tumor. The use of non-optimal boluses can endanger tumor control by under dosing the target [2].

With the arrival and still maturing technology of three-dimensional printing, some studies have already tested and applied the concept of 3D-printed boluses [2, 3], to optimize treatment preparation time and reduce overall costs [2]. Applications of patient-specific 3D-printed bolus are also investigated for range shifter air gap reduction in intensity-modulated proton therapy [4]. All the 3D-printed boluses in these studies have been created by using computed tomography (CT) data.

The usage of radiotherapy planning CT images is not the best method to produce 3D-printed boluses to be fit under an immobilization mask, which is a fixation for the body part to treat, made with thermoplastic material in contact with the patient’s skin. A second CT-scan must often be done with the immobilization mask placed on top of the bolus, thus increasing total dose received by the patient and adding extra workload to the radiotherapy staff (re-planning). Furthermore the quality of the bolus is limited by the relatively poor resolution of the CT-scan and by the immobilization mask modifying the skin outline. These models if not smoothed, can result in a jagged 3D-printed bolus which can be uncomfortable for the patient and cause poor dose irradiation.

When choosing a 3D-surface scanner and related software for image reconstruction, five parameters need to be evaluated: accuracy, resolution, speed, versatility and ease of use. A high accuracy allows for measurements at first draft to be immediately usable, with a perfect design fit without the need of image manipulation and extensive post-processing; a high resolution allows to properly image not only smooth surfaces but also ones with steep curvature, like commonly found on the human body (ear, fingers, etc.); a high image acquisition speed permits high quality tracking for a fast image acquisition, which is very important when scanning humans who move simply by breathing or discomfort, or want to acquire scans in deep inspiration breath hold; a high versatility allows to scan all types of material, colors, etc., so to be used both on humans and on phantoms (for quality assurance tests and other studies); and finally an ease of use that allows for quality results even with non-skilled users because of its simplicity of operation.

Several non-expensive 3D-scanners can be found on the market. One has recently been tested on patients, for other purposes than the production of bolus, and needed a workaround for its use [5] (construction of a gantry support for the 3D-scanner, allowing a restricted angle of scan). Another scanner was tested on a dark phantom, Alderson RANDO® phantom, (The Phantom Laboratory, Salem, NY, USA) which needed to be powder sprayed because the 3D-scanner was not able to scan dark surfaces [6]. To avoid these limitations in this study and take into account accuracy, resolution, speed, versatility and ease of use, a high-end scanner was chosen. This should allow to assure quality performance even for complex anatomy sites as well as to obtain results reproducible, reliable and stable over time.

In this study, we investigate the potential use of a high-grade, high-resolution surface-scanner to produce bolus models to be 3D-printed.

## Methods

First tests were performed on a phantom to verify the superiority of a high-end surface-scanner compared to high resolution CT data for bolus modelling. The head of an Alderson RANDO® phantom (without powder spray) was used to create two different Stereolithography (STL) models of a bolus in the orbital region: bolusCT created from a CT scan and bolusS created from a 3D surface scan. The CT scanner used was a CT Big Bore (Philips, the Netherlands) with a voxel resolution of 0.9 × 0.9 × 0.8 mm^3^ (120 kV, 550 mAs/slice, FOV 350, pitch 0.563). Using Eclipse Treatment Planning System version 13.6 (Varian Medical Systems, USA), a 5 mm thick bolus (bolusCT), was created in the right orbital region of the phantom.

**Fig. 1:**
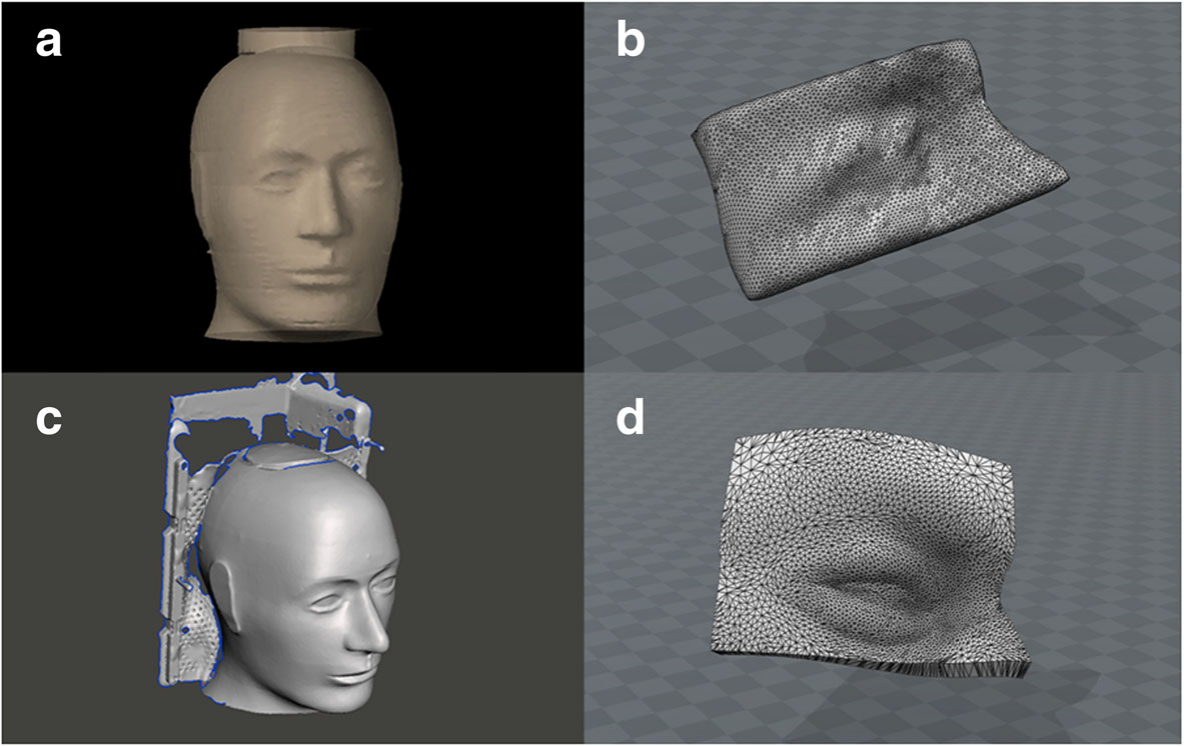
**a** 3D model of the RANDO® phantom using CT-data with its corresponding bolus model (**b**) and 3D model of the phantom using surface-scanner data (**c**) with its corresponding bolus model (**d**)

For bolusS, a portable metrology grade surface-scanner (HandySCAN™ 700, Creaform, Canada) was used to scan the phantom. The resulting surface image was cropped around the right orbital region and extruded to a thickness of 5 mm using the accompanying software VXelements (Creaform, Canada). The surface scan resolution is 0.050 mm. Fig. 1 shows the rendering of the RANDO® head using CT data and the HandySCAN™ 700, as well as the corresponding bolus models. For comparison, a traditional hand-made wax bolus, bolusW, was also created by hand pressing warmed-up wax on the phantom.

**Fig. 2:**
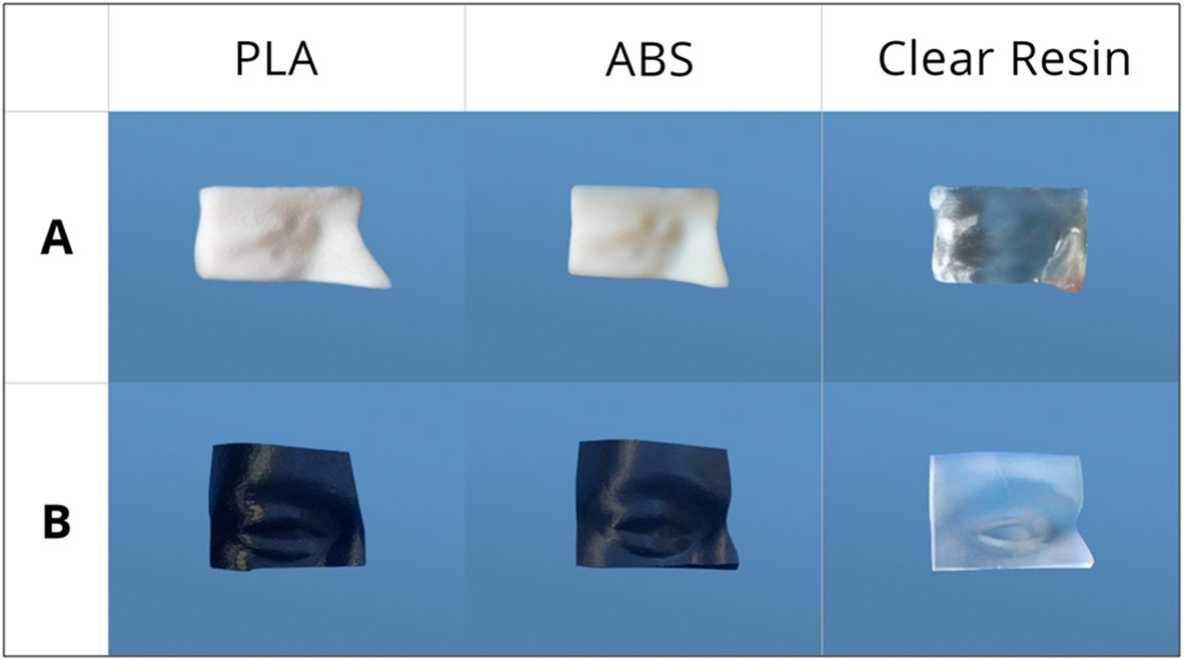
The 3D-printed boluses created for this study. (**a**) The top three boluses are bolusCT based on CT-scan data. (**b**) The bottom three boluses are bolusS based on surface-scanner data. *Abbreviations*: ABS = Acrylonitrile butadiene styrene; PLA = Polylactic acid

To verify that the study was not influenced by the 3D-printing technique and printer resolution and accuracy, bolusCT and bolusS were printed three times using three different printers and materials (Fig. 2): clear photopolymer resin using a stereolithography (SLA) printer (Form 2, FormLabs, USA); digital Acrylonitrile Butadiene Styrene (ABS) using a PolyJet (PJ) printer (Objet260 Connex3, Stratasys); and PolyLactic Acid (PLA) using a fused deposition modelling (FDM) printer (Replicator+, MakerBot, USA). A brief description of the different printing modalities can be found in Michiels et al. [7].

**Fig. 3:**
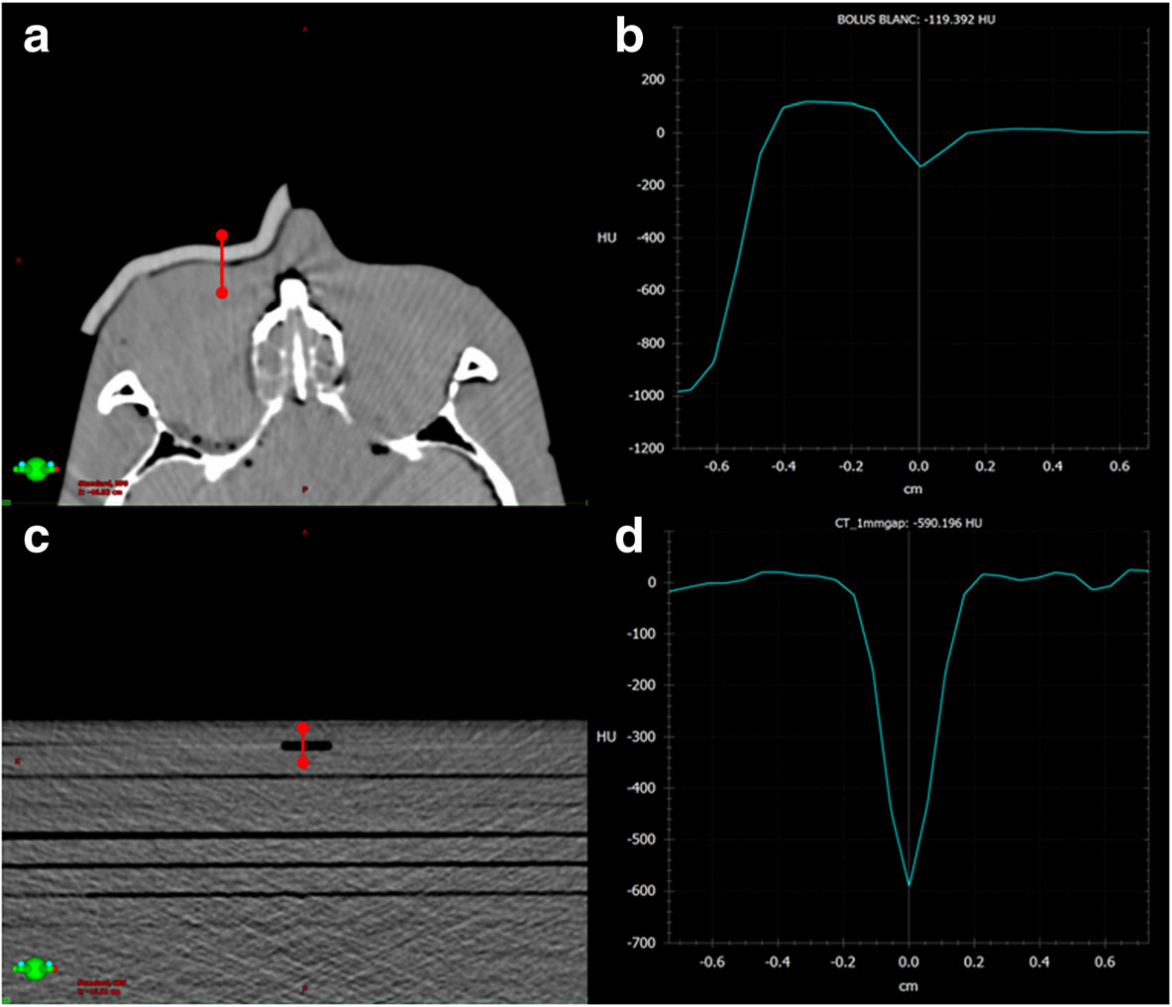
**a** One slice of CT-scan of the RANDO® phantom with the ABS bolusS. **b** HU profile of the air gap between the RANDO® phantom and the ABS bolus. **c** CT-scan of an artificially created air gap of 1 mm. **d** HU profile of the 1 mm air gap

Each 3D-printed bolus as well as the wax bolus was inspected and placed on the phantom head and a CT-scan was performed to investigate the fitting. The CT used the same scanning protocol as mentioned above. To quantify the airgap between the bolus and the phantom surface, profiles of the Hounsfield Unit (HU) at 30 different points of the contact surface were traced on all bolus+phantom images. For each profile, the minimum HU value in the air gap was used to estimate the width of the air gap, Fig. 3a and b.

In order to relate the HU values to air gap distances, artificial air gaps of different widths were created using spacers of 0.6 mm, 1 mm, and 2 mm thickness between water-equivalent slabs (RW3, PTW, Germany). Using the same CT scan protocol, images of these gaps were acquired and HU curves extracted (*n* = 3), Fig. 3c and d. To compare the HU corresponding to the air gap for each bolus, the Wilcoxon signed-rank test and the Mann-Whitney test were used, with *p-values* < 0.05 considered statistically significant.

In the second part of this study, initial tests on healthy volunteers verified that the surface-scanner can be used to image different anatomical areas of the human body.

A total of 10 healthy volunteers, staff members of the department of radiation oncology of Geneva University Hospital, consented to participate in this study. Four out of ten were female volunteers and the participants’ median age was 40 years old (range 23–60 years old). A total of 6 anatomical areas were imaged with the surface-scanner on the volunteers: face, scalp, breast, leg, ear and, hand.

Several points needed to be investigated: the influence of skin reflection on image acquisition, ease of use, and acquisition time. Surface images were acquired with the less expensive HandySCAN™ 300 (Creaform, Canada), which has a resolution of 0.1 mm, 10 times higher than the CT scans. The scanner reconstructs surfaces based on deformation of laser lines which requires calibrating the laser intensity based on the reflectivity of the surface to scan. Its dynamic range was sufficient to scan both the black surface of the RANDO® phantom and slightly transparent human skin.

An automatic reference frame is constructed by placing reflective markers on or around the object to be scanned. They can be glued directly to the skin (or indirectly on medical plaster, that use hypoallergenic glues), which allows a scan to be interrupted and re-started. Multiple scans can also easily be fused if scanned in the same reference frame. The post-processing software VXelements was used to clean the mesh and create the bolus from the acquired surface scan. The bolus is defined by extruding a shell with the required thickness on a selected part of the surface. Once the surface image acquired and bolus modelled, they were 3D printed in-house using PLA on a FDM printer (Ultimaker 3 Extended, Ultimaker, Netherlands). To avoid the use of radiation, the fitting of the boluses was evaluated visually by inspecting bolus-skin contact through purpose-made holes in the boluses.

## Results

Regardless of the 3D scan, material, and printing method used, all 3D printed boluses properly reproduce the shape of the region of interest and the planned thickness. There is, however, already a significant visual difference between the boluses based on CT-scan data and those from the surface scan data. The surface scan based boluses are smoother and fit better on the phantom. Differences are also noticeable between different printers for the same 3D model. The printing technique has the expected result. An FDM created bolus has a slightly rougher texture than the SLA produced resin ones, which feel smoother.

These observations are supported by the measurements of the air gaps. The mean ± SD of the minimum HU value over the 30 air gap profiles taken for all boluses can be found in Table 1. For all three materials used, there is a significant difference in the mean HU between the CT-scan and surface-scanner based boluses (*p-value* < 0.0001) in favor of the surface-scanner models.

**Table 1:** Hounsfield Units measurements of air gaps between boluses and RANDO® phantom external surface

MATERIALS	Mean HU	Standard deviation HU	Maximum Gap Depth HU	Maximum Gap Depth (mm)
BolusCT				
Clear Resin	− 295	58	− 627	1–2
Digital ABS	− 387	148	− 590	1–2
PLA	− 390	102	− 591	1–2
All	− 383	134	−627	1–2
BolusS				
Clear Resin	− 45	51	− 167	< 0.6
ABS	− 191	54	− 313	< 0.6
PLA	− 155	77	− 294	< 0.6
All	− 130	87	−313	< 0.6
BolusW				
Wax	−469	219	− 860	2–3

The mean ± SD HU of the air gaps for the wax bolus was − 469 ± 219 HU, revealing a greater variation in air gap along the surface of the wax bolus. There is no statistical difference when comparing the air gaps between the wax and CT-scan based boluses (*p-value* = 0.0645).

As can be seen in Table 1, the maximum air gap in HU is smaller by nearly a factor of 2 for bolusS with respect to bolusCT, regardless of the material.

The calibration air gaps of 2 mm, 1 mm and 0.6 mm correspond to mean ± SD HU of − 876 ± 5, − 586 ± 4 and − 463 ± 3 respectively, implying that mean gaps for all boluses were ≤ 0.6 mm. The maximum air gaps however were of the order of 2 mm for bolusW, of 1-2 mm for bolusCT and, smaller than 0.6 mm for bolusS.

**Fig. 4:**
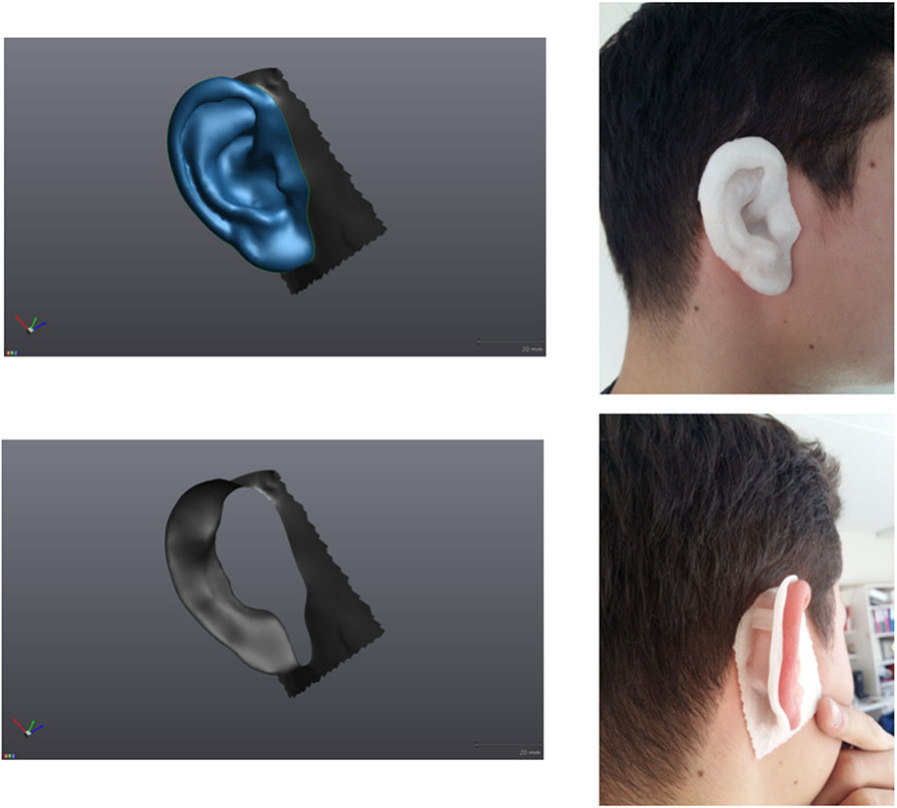
Left: Surface model of an ear split in 2 parts. Right: the 3D-printed boluses fitting the ear of a volunteer

**Fig. 5:**
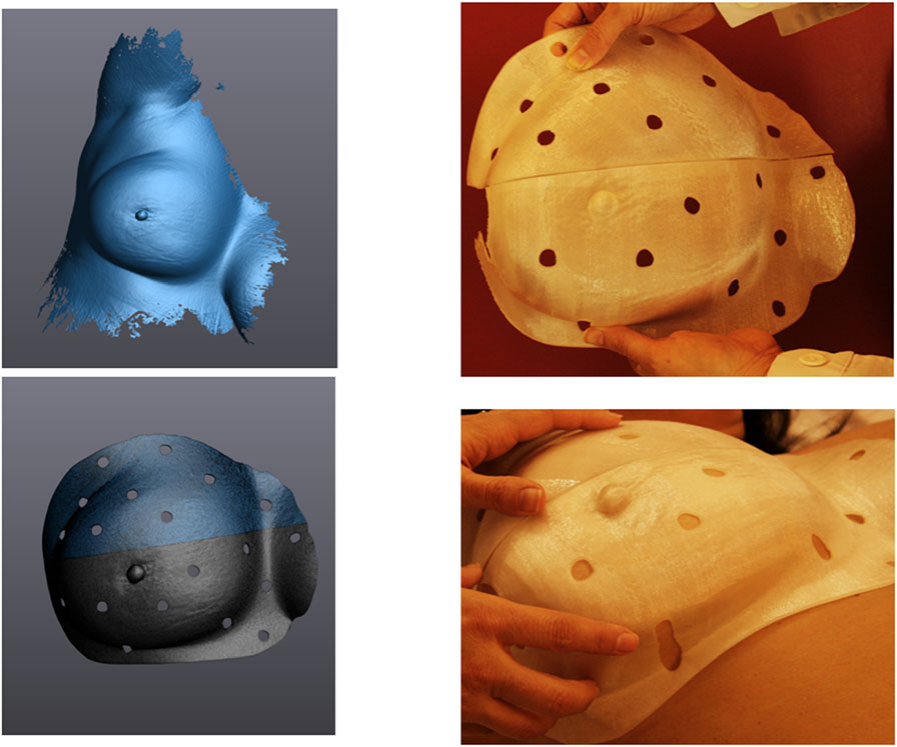
Left, from top to bottom: Original acquired surface model of a breast; Bolus region cropped and divided in 2 subparts: upper part and lower part. Right: The 3D-printed boluses fitting the breast of the volunteer

**Fig. 6:**
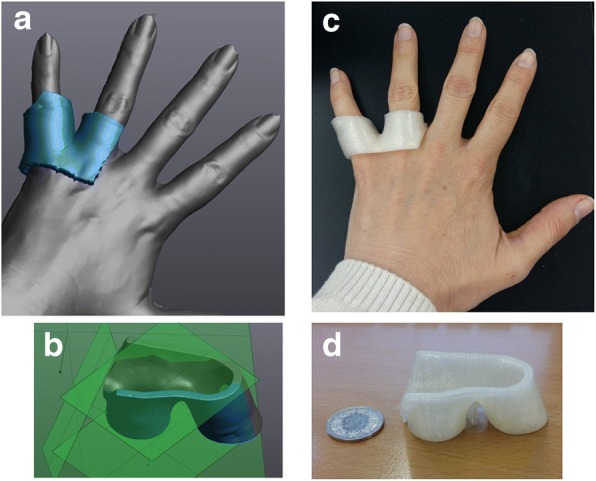
**a** Creating the 3D model for the hand bolus using surface-scan data of a hand by extruding the region of interest. **b** Trimming the bolus model using flat planes on VXElements. **c** Testing the fit of the bolus on the subject’s hand. **d** Final 3D-printed bolus of the 4th and 5th knuckle

The results of the preliminary tests on healthy volunteers show that 3D printed boluses based on surface-scanner data do generate a proper, comfortable fit and correctly reproduce the anatomical form. Two examples are shown in Fig. 4. A large breast bolus, Fig. 5, not only fitted comfortably on the breast but also had a good contact bolus-skin visible via visual inspection through the holes made in the bolus. To fit this bolus in the printer, as well as to easily fit it on the volunteer’s skin, the model was split and printed in two parts. Figure 6 shows the fitting of a bolus created around the fingers of a hand.

The time required to acquire a proper image of the skin surface depends on the anatomical site and ranged for our preliminary tests from 7 min (ear) to less than 1 min for the large breast in Fig. 5. Using deep inspiration breath hold, a surface-scanner image of a breast was also acquired within 40 s (the bolus fit was also correct). The ease with which a scan can be completed depends on the clearance for the scanner to move around the body part to scan and the complexity of its form, but not the speed of image acquisition because we use a fast measuring device (205,000 measurements/s). Time to post-process the surface image is also anatomical site dependent. Complex shapes are more time consuming as they usually may contain small holes and other scanner artifacts that need to be corrected first. For smoother surfaces, like the breast of Fig. 5, post-processing clean-up, region selection and model creation only took a few minutes. Figure 6 shows the modeling of a hand to create a bolus around 2 fingers. Though the post processing was not rapid, defining the planes of cut for the mesh around the hand, the first draft was immediately usable without any need of enlarging or shrinkage of the surface to make the bolus fit the hand. All 3D-printed bolus using surface-scanner models did not require enlarging or shrinking of the initial model acquired in-vivo.

## Discussion and conclusion

A surface-scanner was successfully used on a phantom to create a 3D-printed bolus which showed superior fitting compared to a 3D-printed bolus based on CT-scan. Regardless of the 3D printing method, or material used, the maximum air gaps of the boluses based on high-resolution surface scans were always less than 0.6 mm.

3D-printed bolus HU profiles show that CT-scan based boluses had greater air gaps than those of the surface-scanner based ones. This is not surprising because, when using CT data, the voxel size is fixed so that CT resolution is too low in regions of high curvature.

The wax bolus was not statistically different compared to the 3D-printed bolusCT, but this might not be the case in real clinical cases. Indeed, the wax bolus in this study was produced on a rigid, hard phantom. For a real patient, the bolus might be of lower quality because of the difficulties to press and shape the wax on soft tissue, especially if it is also damaged by the tumor. Therefore, 3D-printed boluses created from CT data in clinic are likely superior to hand-made ones, as confirmed by Canters et al. [2] in case of electron treatments.

Though such a good skin-bolus contact (air gaps < 0.6 mm as obtained with the BolusS) might not be necessary from a dosimetric point of view for typical large X-rays fields of 3D conformal treatment techniques, it might be advantageous when using intensity modulated radiation therapy and volumetric arc techniques that use small beamlets of radiation. In fact, the dose under the bolus decreases from planned doses in the presence of air gaps, deviations increasing with increasing angle of incidence of the beam radiation and decreasing beam’s field-size [8] (4% dose reduction for a 8 × 8 cm^2^ field size, an angle of incidence of 45° and a 4 mm air gap).

This methodology is easily applicable to photon beam therapy, with and without immobilization masks. In photon beam radiotherapy the bolus thickness can often be decided before the CT scan [3] and a 5 mm thick bolus is generally sufficient to obtain a good coverage of superficial target using intensity modulated treatments. In fact, targets in these irradiation techniques are generally cropped at 5 mm under the skin surface to prevent dosimetric optimization problems, as discussed by Verbakel et al. [9]. Therefore, adding a 5 mm bolus would position a superficial tumor sufficiently in depth to obtain a correct plan optimization and dose delivery.

The use of surface scanner for bolus creation is more difficult in the case of particle therapy when we do not know a priori what radiation modality (beam energy) and beam direction will be applied. Furthermore, if one wants to modulate the beam’s penetration depth using a varying bolus thickness, as suggested by Su et al. [10] for electron therapy, then the bolus can only be designed after the target volume has been drawn on CT scan result. Therefore the methodology presented in our study can be considered primarily designed for megavoltage X-rays radiotherapy.

Another important advantage of having a bolus printed prior-CT simulation is the possibility to take into account the real final density and homogeneity of the bolus used. Any imperfection could be accounted for at planning, increasing the chances of a dose delivery similar to the planned one, or put in evidence the need to re-print the object before treating the patient. In fact in 3D-printing of clinical boluses, one also has to pay attention to the fill factor [11], printing speed and structure, as these will influence the final density considerably (even if set to 100% in the case of FDM printers), as well as the radiological properties of the material used [7, 12–15]. According to a recent publication by Craft et al. [14] every 3D-printed object should be CT scanned to verify proper quality in terms of HU before use.

To assure correct dose calculations in the treatment planning system, the HU taken from the planning CT scans have to be accurate. Radiotherapy CT scanners are “calibrated” on water-equivalent materials (human body) and the HU can be wrong for other materials. This can represent a problem for plastic boluses not water-equivalent, if they are present on the patients during the CT [13]. For these reasons, PLA and ABS material were previously tested [16] and used in this study as their radiological properties work well for planning [12] and do not need bolus contouring. Test on volunteers used only PLA, which was considered our material of choice for future clinical studies. One solution for non-water-equivalent material is to override the HU of the bolus structure in the TPS. This requires extra contouring time to define the bolus before dose calculation. This task can probably be made faster by either fusing a separate CT-scan of the bolus by itself (with the bolus structure easily segmented with automatic tools) or importing the actual 3D-model of the bolus into the TPS. Unfortunately this is currently not yet possible in most TPS systems. To solve this issue in the future and to permit easy inclusion of 3D surface scans in radiotherapy workflows, TPS vendors should add options to import surface models into their software. This would allow the integration in the treatment planning process of 3D-scanner images for bolus models. Working groups are already dealing with the DICOM standardization of surfaces images and 3D-printing STL models for patient care, patient imaging records, data exchange, etc. [17].

There are other clear benefits of using a high-end surface-scanner on humans. Although tests were performed by little experienced users, images were acquired with small artifacts, if none, depending on the anatomical site imaged, requiring no additional scans and post-processing, thereby saving time. The 3D-scanner tested also uses optical reflectors, which eliminates the need for a rigid setup and a 3D-scanner gantry, and allows for dynamic referencing, meaning the object or patient scanned can move without altering the quality of the scan. Furthermore, because the surface-scanner is used freehand, the patient is not restricted to a specific position, improving patient anatomy scan “access” and comfort. The scan can be stopped and continued, which would be impossible using a consumer-grade scanner such as the one described by *Sharma* et al. [5]. It is possible with the high-end 3D-scanner tested to acquire images of large anatomical sites (e.g. a leg or large breast, Fig. 5) without being restricted by the scanner’s field of view or elaborating a complex gantry surface-scanner support where the table is mobile, as suggested by *Sharma* et al. [5].

Finally, first results on volunteers show a good fit of all tested bolus obtained from surface-scanner models because of no appreciable image deformation and the high accuracy of the system tested. This aspect together with 3D-scanner image acquisition requiring generally 2 min (max 7 min) will allow the integration of such a workflow in routine.

Based on the results of this study, the use of a high-grade surface-scanners to create prior to CT simulation 3D-printed boluses for megavoltage X-rays radiotherapy in clinical routine looks promising to improve radiotherapy workflow, patient comfort and treatment quality.

## Abbreviations

ABS: Acrylonitrile butadiene styrene
bolusCT: Bolus made from CT data
bolusS: Bolus made from surface-scanner data
bolusW: Bolus made by hand directly on the phantom with wax
CT: Computed Tomography
DICOM: *Digital Imaging and Communications in Medicine* file format
FDM: Fused deposition modeling
HU: Houndsfield Unit
PJ: Polyjet
PLA: Polylactic acid
SD: Standard deviation
SLA: Stereolithography printer method
STL: Stereolithography
TPS: Treatment Planning System
